# Geographic and Viral Etiology Patterns of *TERT* Promoter and *CTNNB1* Exon 3 Mutations in Hepatocellular Carcinoma: A Comprehensive Review

**DOI:** 10.3390/ijms26072889

**Published:** 2025-03-22

**Authors:** Mariana Leonardo Terra, Thaís Barbosa Ferreira Sant’Anna, José Junior França de Barros, Natalia Motta de Araujo

**Affiliations:** Laboratory of Molecular Virology and Parasitology, Oswaldo Cruz Institute, Oswaldo Cruz Foundation (FIOCRUZ), Rio de Janeiro 21040-900, Brazil; marianaterra.biomed@gmail.com (M.L.T.); thaisbfsantanna@gmail.com (T.B.F.S.); barros@ioc.fiocruz.br (J.J.F.d.B.)

**Keywords:** hepatocellular carcinoma, TERT promoter, CTNNB1, hepatocarcinogenesis, HBV, HCV, somatic mutations

## Abstract

Hepatocellular carcinoma (HCC) is the most common primary liver malignancy and a leading cause of cancer-related mortality worldwide. Genetic alterations play a critical role in hepatocarcinogenesis, with mutations in the telomerase reverse transcriptase promoter (*TERTp*) and *CTNNB1* exon 3 representing two of the most frequently reported somatic events in HCC. However, the frequency and distribution of these mutations vary across geographic regions and viral etiologies, particularly hepatitis B virus (HBV) and hepatitis C virus (HCV). This study aimed to assess the global distribution and etiological associations of *TERTp* and *CTNNB1* exon 3 mutations in HCC through a comprehensive literature review. Our analysis, encompassing over 4000 HCC cases, revealed that *TERTp* mutations were present in 49.2% of tumors, with C228T being the predominant variant (93.3% among mutated cases). A striking contrast was observed between viral etiologies: *TERTp* mutations were detected in 31.6% of HBV-related HCCs, compared to 66.2% in HCV-related cases. *CTNNB1* exon 3 mutations were identified in 23.1% of HCCs, showing a similar association with viral etiology, being more common in HCV-related cases (30.7%) than in HBV-related tumors (12.8%). Geographically, both mutations exhibited comparable patterns, with higher frequencies in Europe, Japan, and the USA, while lower rates were observed in China, Taiwan, and South Korea. Our findings underscore the distinct molecular profiles of HCC according to viral etiology and geographic origin, highlighting the need for region- and etiology-specific approaches to HCC prevention, diagnosis, and targeted therapy.

## 1. Introduction

Hepatocellular carcinoma (HCC) is the most common type of primary liver cancer, representing approximately 75–85% of all liver cancer cases. Globally, HCC ranks as the sixth most commonly diagnosed cancer and the third leading cause of cancer-related deaths, with an estimated 866,000 new cases and 758,000 deaths annually [1,2]. The incidence of HCC varies geographically, with the highest rates observed in East Asia and sub-Saharan Africa, primarily due to the high prevalence of chronic hepatitis B virus (HBV) infection in these regions [3]. Despite advances in medical treatments, the prognosis for HCC remains poor, with a 5-year survival rate of less than 20% [4]. Early diagnosis is crucial for improving outcomes, but many cases are detected at advanced stages due to the asymptomatic nature of early HCC and limited screening in high-risk populations [5].

**Figure 1:**
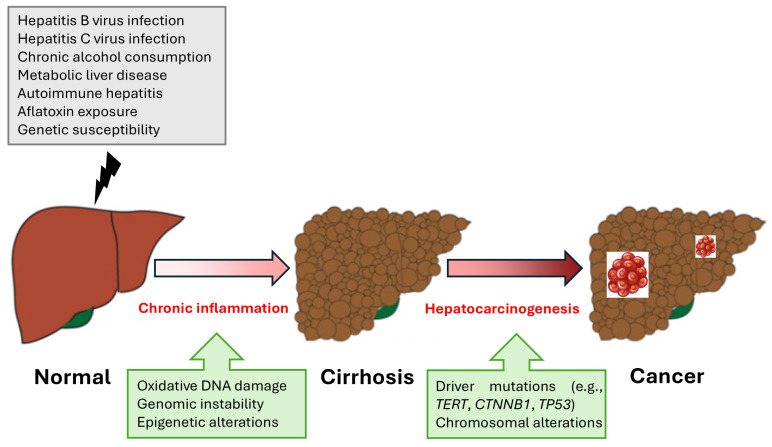
Mechanisms of hepatocarcinogenesis from chronic liver disease to cancer. The progression from normal liver to cirrhosis and HCC is driven by chronic liver injury. HBV and HCV infections, chronic alcohol consumption, metabolic dysfunction-associated steatotic liver disease (MASLD), autoimmune hepatitis, aflatoxin exposure, and genetic susceptibility contribute to persistent inflammation, fibrosis, and cirrhosis. Chronic inflammatory processes induce oxidative DNA damage, genomic instability, and epigenetic alterations, leading to the accumulation of key driver mutations (e.g., *TERT*, *CTNNB1*, and *TP53*) and chromosomal aberrations. These molecular changes facilitate hepatocyte transformation, uncontrolled proliferation, and malignant progression, ultimately resulting in HCC.

Several risk factors contribute to the development of HCC. Chronic infection with HBV or hepatitis C virus (HCV) is the most significant risk factor, accounting for about 76% of HCC cases worldwide [6]. Chronic alcohol consumption is another major risk factor, leading to cirrhosis and subsequently increasing the risk of HCC. Metabolic dysfunction-associated steatotic liver disease (MASLD), formerly known as NAFLD, has emerged as an increasingly important risk factor for HCC, driven by the global rise in obesity, type 2 diabetes, and metabolic syndrome. Additionally, exposure to aflatoxins, toxins produced by certain fungi found in food, has been linked to HCC development [4,7]. Genetic factors and a family history of liver cancer also play a crucial role in the susceptibility to HCC, highlighting the need for personalized approaches in risk assessment and management [8,9]. The pathophysiology of HCC is complex and involves a multistep process of liver carcinogenesis. Chronic liver injury, regardless of the underlying cause, leads to persistent inflammation, hepatocyte death, and regeneration, which, over time, can result in liver fibrosis and cirrhosis. Cirrhosis is a significant precursor for HCC, with studies showing that 80–90% of HCC cases develop in the context of cirrhosis [4]. The progression from cirrhosis to HCC is a protracted process in which the liver’s regenerative attempts drive genetic and epigenetic alterations, fostering a pro-carcinogenic microenvironment. This environment is marked by the activation of various signaling pathways that drive cellular proliferation, angiogenesis, and resistance to apoptosis, all of which contribute to the malignant transformation of hepatocytes [10] (Figure 1).

Among the numerous genetic and molecular alterations implicated in HCC, mutations in the telomerase reverse transcriptase (*TERT*) promoter (*TERTp*) and catenin beta-1 (*CTNNB1*) genes are particularly noteworthy [11]. Other genetic alterations, such as mutations in *TP53*, *AXIN1*, and *ARID1A*, are also frequently observed in HCC, reflecting the molecular heterogeneity of the disease [12]. In HCC driven by viral infections (HBV and HCV), *TERTp* mutations are the most frequent genetic alteration [13], leading to the upregulation of telomerase activity, which allows cancer cells to maintain telomere length and achieve replicative immortality [14]. Mutations in *CTNNB1*, which encodes β-catenin, a key component of the Wnt signaling pathway, are also observed in virally induced HCC, although with a lower prevalence compared to non-viral-associated cases [15]. The frequency of these mutations can vary across different geographic regions and may reflect underlying differences in viral genotype, host factors, and environmental exposures [16,17,18]. There is some concordance between *TERTp* and *CTNNB1* mutations in HCC, suggesting a potential cooperative role in hepatocarcinogenesis, as studies indicate their co-occurrence may contribute to tumor development and progression [13,19]. This concordance highlights the importance of these genetic alterations in the pathogenesis of HCC and suggests potential targets for therapeutic intervention [16].

Given the significant global burden of HCC and the critical role of *TERTp* and *CTNNB1* mutations, understanding the frequency and distribution of these mutations, as well as their interactions, is essential for developing more effective diagnostic and therapeutic approaches. In this review, we synthesize the current literature on the frequency of *TERTp* and *CTNNB1* exon 3 mutations in HCC, with a focus on geographic variation and viral etiologies. We also examine the complex interactions between these mutations and their impact on tumor progression.

## 2. Structure and Function of Telomeres and Telomerase

Telomeres are specialized structures located at the ends of linear chromosomes, playing a critical role in maintaining genomic stability and protecting the ends of chromosomes from degradation and fusion. Structurally, telomeres are composed of repetitive nucleotide sequences, typically rich in guanine, such as the hexanucleotide repeat sequence (TTAGGG) in vertebrates, which can be repeated thousands of times [20]. These sequences are bound by a complex of proteins known as the shelterin complex, which helps to form a protective cap. This complex includes proteins such as TRF1, TRF2, POT1, TIN2, TPP1, and RAP1, each playing specific roles in telomere maintenance and protection [21]. The telomere structure can form a T-loop, where the single-stranded telomeric DNA folds back and inserts itself into the double-stranded telomeric region. This configuration stabilizes the ends of chromosomes and prevents them from being mistakenly recognized as DNA breaks [22]. This structural configuration is crucial for maintaining the integrity and functionality of chromosomes during cell division.

Telomerase is a ribonucleoprotein enzyme that adds telomeric repeats to the ends of chromosomes, thereby counteracting the progressive shortening that occurs with each round of DNA replication. Telomerase comprises two essential components, the telomerase reverse transcriptase (TERT) protein and the telomerase RNA component (TERC), which serves as a template for the addition of new telomere sequences [23]. Telomerase activity is tightly regulated and predominantly active in stem cells, germ cells, and certain white blood cells, while most somatic cells exhibit little to no telomerase activity, leading to gradual telomere shortening and eventual replicative senescence [24]. The enzyme’s ability to elongate telomeres is vital for cells that need to divide extensively and maintain their proliferative capacity over long periods. Dysregulation of telomerase activity can lead to diseases associated with telomere shortening, such as dyskeratosis congenita and idiopathic pulmonary fibrosis, highlighting its importance in cellular homeostasis and organismal health [25].

The *TERT* gene, located on chromosome 5p15.33, encodes the catalytic subunit of TERT. The regulation of *TERT* expression and activity is complex, involving multiple layers of control at the transcriptional, post-transcriptional, and post-translational levels. Canonically, transcriptional regulation of *TERT* is mediated by various factors, including c-Myc, which acts as a positive regulator, and repressive elements such as the WT1 transcription factor [26,27]. Additionally, alternative splicing of *TERT* mRNA can produce isoforms with distinct functions, further adding to the regulatory complexity [28]. Non-canonical pathways of *TERT* involve its roles beyond telomere lengthening. TERT has been found to participate in mitochondrial function, protection against apoptosis, and regulation of gene expression independent of its telomerase activity [29,30]. In mitochondria, TERT can improve cellular resistance to oxidative stress and maintain mitochondrial DNA integrity. The protein can also interact with signaling pathways involved in cellular stress responses, such as the NF-κB pathway, influencing inflammation and cell survival mechanisms [31]. Understanding both canonical and non-canonical pathways of *TERT* is essential for comprehending its full spectrum of biological functions and its implications in aging, regenerative medicine, and cancer.

## 3. The Role of *TERT* in HCC

*TERT* plays a critical role in the development and progression of various cancers, including HCC. In the context of HCC, *TERT’*s function extends beyond its canonical role in telomere maintenance, significantly contributing to tumorigenesis through multiple mechanisms. The overexpression of *TERT* in HCC is often driven by mutations in *TERTp*, leading to its reactivation in somatic cells where it is normally silent [32]. *TERTp* mutations in HCC predominantly occur at two hotspots involving cytosine to thymine transitions: 1295,228 C>T (C228T, also referred to as -124C>T) and 1295,250 C>T (C250T, also referred to as -146C>T). These mutations are located 124 and 146 base pairs upstream of the *TERT* transcription start site, respectively, and are among the most frequent genetic alterations observed in HCC [19]. These alterations result in the creation of de novo binding sites for ETS transcription factors, particularly GABP, which leads to increased transcription of *TERT* and subsequent telomerase activity [14]. The upregulation of *TERT* is a critical factor in the immortalization of hepatocytes, allowing them to evade replicative senescence and accumulate further genetic mutations that drive malignant transformation. Enhanced telomerase activity maintains telomere length, permitting continuous cell division without compromising genomic integrity, which is crucial for tumor growth [33].

The presence of *TERTp* mutations has significant clinical implications for HCC. These mutations are not only crucial for the pathogenesis and progression of HCC but also play a pivotal role in the clinical management, prognosis, and potential therapeutic targeting of the disease. *TERTp* mutations are strongly associated with poor prognosis in HCC patients. Several studies indicate that patients harboring these mutations often exhibit shorter overall survival and higher recurrence rates compared to those without the mutations. This is particularly evident in patients with advanced stages of HCC, where the presence of *TERTp* mutations correlates with more aggressive tumor behavior and greater metastatic potential [17,34]. Moreover, studies have shown that HCV-related HCCs have a higher frequency of *TERTp* mutations compared to HBV-related HCCs [13,35], reflecting the distinct oncogenic mechanisms of each virus and the different pathways driving liver cancer development.

Beyond promoting cellular proliferation, TERT also plays a crucial role in modulating the tumor microenvironment. TERT can activate several signaling pathways that are pivotal in cancer biology. For instance, TERT has been shown to interact with the NF-κB pathway, which is known to regulate inflammation and immune responses [31]. Through this interaction, TERT can create a pro-inflammatory environment that supports tumor growth and progression. Inflammatory cytokines produced in this environment can promote angiogenesis, providing the tumor with the necessary blood supply for its growth. Additionally, TERT can influence the Wnt/β-catenin pathway, further promoting cellular proliferation and survival [36,37]. Dysregulation of the Wnt/β-catenin signaling is a common feature in HCC and contributes to tumor progression and metastasis.

Telomerase-based cancer therapeutics represent a promising frontier in oncology, since it is highly expressed in various cancer types but not in most somatic cells, making it an attractive therapeutic target [38]. Recent advancements include the development of telomerase inhibitors such as imetelstat, a first-in-class oligonucleotide that binds to the RNA template of telomerase, inhibiting its activity and inducing telomere shortening, leading to cancer cell apoptosis [39]. It exhibits potent activity in preclinical models of various cancers, including liver cancer [40], although its clinical development has been more advanced in hematologic malignancies [41]. Beyond small-molecule inhibitors, immunotherapeutic approaches targeting TERT are being developed. TERT-targeting vaccines, such as GV1001 and GRNVAC1, aim to stimulate the immune system to recognize and attack telomerase-expressing tumor cells [42,43]. These vaccines have shown promising results in early-phase trials for other malignancies, and efforts are being made to overcome the immunosuppressive tumor microenvironment of HCC, which often limits vaccine efficacy. One strategy being explored is the combination of TERT vaccines with immune checkpoint inhibitors (ICIs) to enhance antitumor immune responses [44]. Additionally, gene-editing technologies such as CRISPR/Cas9 provide a novel approach to directly disrupt telomerase expression in tumor cells. Preclinical studies have demonstrated that CRISPR-mediated *TERT* knockout in HCC cell lines leads to significant reductions in proliferation and increased apoptosis, but challenges remain regarding delivery mechanisms, off-target effects, and long-term safety [45,46]. While telomerase inhibition represents an attractive therapeutic avenue, several challenges remain. *TERTp* mutations are early oncogenic events, meaning that by the time HCC is diagnosed, tumors have often developed alternative survival pathways, reducing the standalone efficacy of telomerase-targeting therapies [13]. Additionally, telomerase is active in certain stem cell populations, raising concerns about potential long-term toxicities in regenerative tissues such as the bone marrow and gastrointestinal epithelium [47]. As a result, combinatorial approaches that integrate telomerase inhibition with other targeted therapies, such as Polo-like kinase 1 (PLK1) inhibitors, may be necessary to achieve sustained tumor control in HCC. Recent studies have demonstrated that HCC cells harboring *TERTp* mutations are more sensitive to PLK1 inhibitors, suggesting that combining telomerase inhibition with PLK1-targeted therapy could enhance treatment efficacy in these tumors [48].

The detection of *TERTp* mutations has the potential to serve as a valuable diagnostic and predictive biomarker in HCC. These mutations are early events in hepatocarcinogenesis, and their presence may help in the early detection of HCC, even before clinical symptoms become apparent [13]. Additionally, the presence of these mutations could predict the tumor’s response to certain treatments, enabling more personalized and effective therapeutic strategies [17]. Recent studies have continued to explore the implications of *TERTp* mutations in HCC. For instance, emerging data suggest that these mutations can be detected in circulating tumor DNA (ctDNA), offering a non-invasive method for early diagnosis and monitoring of disease progression [49]. The potential for ctDNA to provide real-time insights into tumor dynamics represents a significant advancement in the field of HCC diagnostics and personalized medicine. Moreover, advancements in next-generation sequencing (NGS) technologies have improved the sensitivity and specificity of detecting *TERTp* mutations, making it feasible to integrate these biomarkers into clinical practice for better prognostic assessments and tailored treatment approaches [50]. This integration could enhance the ability to stratify patients based on their molecular profiles, potentially improving outcomes through more targeted therapeutic interventions.

## 4. Frequency and Geographic Distribution of *TERTp* Mutations in HCC

To gain a broad understanding of the distribution of *TERTp* mutations in HCC, we conducted a comprehensive review of published data by searching PubMed using the keywords “TERT”, “promoter”, “mutations”, and “hepatocellular carcinoma”. Table 1 summarizes the findings from these studies, highlighting the prevalence of the two most common *TERTp* hotspot mutations, C228T and C250T, across different geographic regions and viral etiologies (Table 1).

**Figure 2:**
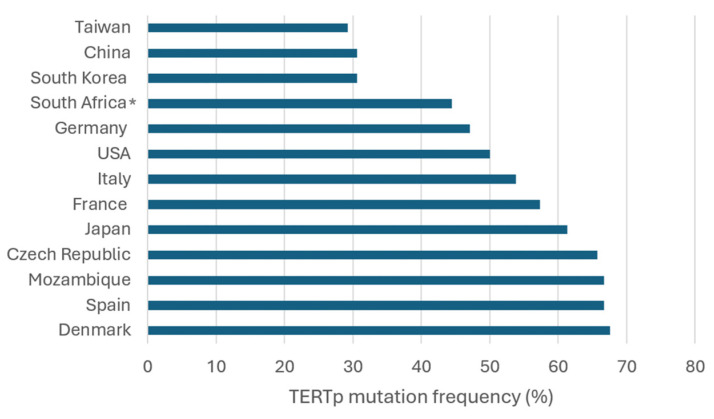
Frequency of *TERT* promoter mutations in HCC cases across different geographic regions. *: Combined data from South Africa, Swaziland, Lesotho, and Transkei.

Analysis of 4133 HCC samples revealed that 49.2% harbored *TERTp* mutations. Among mutated samples, the C228T mutation was predominant, detected in 93.3% of cases, whereas C250T was identified in only 4.9%. These findings underscore the marked predominance of C228T as the principal *TERTp* mutation in HCC. Geographic variations were notable. In Europe, mutation frequencies were generally high. Schulze et al. (2015) [15] reported a frequency of 62.1% in France. Similarly, frequencies of 58.7% and 58.1% were observed in two large studies (n = 305 and n = 759, respectively) [13,51], suggesting that *TERTp* mutations are a consistent feature of French HCC cases. In Italy, mutation frequencies showed substantial variation, ranging from 28.6% in a small cohort of 21 patients [17] to 69.3% in a larger study [52]. The heterogeneity in Italian data might reflect differences in regional patient characteristics or study methodologies. A frequency of 66.7% was found in Spain, although the sample size was particularly limited (n = 9), requiring cautious interpretation [15]. Germany exhibited intermediate levels, with Quaas et al. (2014) [53] reporting 47.4%, while another study (n = 7) described a frequency of 42.9% [54]. Denmark had a high prevalence of 67.6% [55]. In the Czech Republic, Ambrozkiewicz et al. [56] described a similarly elevated frequency of 65.7%. In Asia, mutation frequencies exhibited substantial heterogeneity. Totoki et al. (2014) [19] reported 59.9% in Japan based on an extensive cohort (n = 374). Additional Japanese studies ranged from 36.4% to 81.8% [54,57,58,59,60,61,62], suggesting potential regional or clinical differences within the country. In South Korea, Lee et al. (2016) [63] found 39%, while Jang et al. (2021) [64] observed a lower frequency of 27.8% in a larger cohort (n = 205), indicating potential differences in patient characteristics or screening practices. Taiwan had a relatively low frequency of 29.2% [65]. In China, Yuan et al. (2017) [66] and Yang et al. (2016) [67] reported similar frequencies of approximately 30%, based on robust sample sizes (n = 190 and n = 275, respectively). The higher mutation frequency observed in Japan compared to China, Taiwan, and South Korea may partially reflect differences in viral etiology, as HCV is more prevalent in the former, whereas HBV predominates in the latter regions [35]. In the Americas, mutation frequencies in the USA varied widely. Totoki et al. (2014) [19] found 37.1%, while Chianchiano et al. (2018) [68] reported 71.4%. Killela et al. (2013) [69] described an intermediate frequency of 44.3%. The variability in data may reflect population heterogeneity or different underlying liver disease etiologies. Data from Africa were limited, with Cevik et al. (2015) [54] documenting a frequency of 66.7% in Mozambique, although the sample size was very small (n = 6). Combining data from Swaziland (currently known as Eswatini), Lesotho, Transkei, and South Africa yields a frequency of 44.4% (four mutations in nine samples). However, these results should be interpreted with caution due to the small sample sizes and the overall underrepresentation of African populations in *TERTp* mutation studies (Table 1) (Figure 2).

Comparing viral etiologies, *TERTp* mutations were detected in 31.6% of HBV-associated HCCs, whereas the frequency was substantially higher (66.2%) in HCV-associated cases. Notably, Totoki et al. (2014) [19] reported a 37.4% mutation frequency in HBV-positive Japanese patients and a much higher rate of 74.8% in HCV-positive patients. Similarly, Nishida et al. (2018) [62] observed 51.9% in HBV-related cases and 78.7% in HCV-related HCC in Japan. In Italy, Pezzuto et al. (2016) [70] found mutation rates of 41.7% in HBV-positive and 53.6% in HCV-positive patients. However, two other Italian studies reported exceptions, with higher mutation frequencies in HBV cases compared to HCV: 70% vs. 40% [15], and 66.7% vs. 46.2% [71]. Similarly, in a Spanish cohort, *TERTp* mutations were observed in 100% of HBV-positive cases and 80% of HCV-positive cases [15]. Such discrepancies could reflect the small sample sizes involved, differences in patient selection criteria, or specific regional characteristics. In the USA, Chianchiano et al. (2018) [68] detected *TERTp* mutations in 90% of HCV-associated cases, while none were found among HBV-positive patients (Table 1). The consistently higher prevalence of *TERTp* mutations in HCV-associated HCC aligns with the hypothesis that the chronic inflammation and oxidative stress induced by HCV infection contribute to genomic instability, thereby favoring the acquisition of *TERTp* mutations [72]. In contrast, HBV-associated HCC shows greater variability in *TERTp* mutation frequencies, likely due to differences in HBV genotypes, viral integration patterns, and additional risk factors such as aflatoxin exposure or metabolic disorders [73,74]. These findings suggest that distinct molecular mechanisms drive hepatocarcinogenesis in HBV and HCV infections, with *TERTp* mutations playing a more prominent role in HCV-related tumorigenesis.

**Table 1:** Distribution of *TERT* promoter mutations in HCC across viral etiologies and geographic regions.

Refs.	Country/Region	*TERTp* Mut (n, %) ^a^	C228T (n, %) ^b^	C250T (n, %) ^c^	HBV + Mut (n, %) ^d^	HCV + Mut (n, %) ^e^
[66]	China (n = 190)	57/190 (30)	50/57 (87.7)	7/57 (12.3)	50/153 (32.7)	NA
[67]	China (n = 275)	85/275 (30.9)	84/85 (98.8)	1/85 (1.2)	78/259 (30.1)	NA
[75]	China (n = 35)	11/35 (31.4)	9/11 (81.8)	2/11 (18.2)	NA	NA
[56]	Czech Republic (n = 67)	44/67 (65.7)	41/44 (93.2)	3/44 (6.8)	NA	NA
[55]	Denmark (n = 34)	23/34 (67.6)	23/23 (100)	NA	NA	4/5 (80)
[15]	France (n = 193)	120/193 (62.1) ^f^	106/120 (88.3)	5/120 (4.2)	10/24 (41.7)	27/36 (75)
[13]	France (n = 305)	179/305 (58.7)	168/179 (93.9)	11/179 (6.1)	26/67 (38.8)	49/68 (72.1)
[17]	France (n = 75)	23/75 (30.7)	19/23 (82.6)	4/23 (17.4)	NA	NA
[51]	France (n = 759)	441/759 (58.1) ^f^	404/441 (91.6)	19/441 (4.3)	NA	NA
[54]	Germany (n = 7)	3/7 (42.9)	2/3 (66.7)	1/3 (33.3)	1/3 (33.3)	NA
[53]	Germany (n = 78)	37/78 (47.4)	37/37 (100)	0/37 (0)	NA	NA
[52]	Italy (n = 114)	79/114 (69.3)	79/79 (100)	0/79 (0)	6/10 (60)	72/99 (72.7)
[70]	Italy (n = 127)	64/127 (50.4)	62/64 (96.9)	2/64 (3.1)	5/12 (41.7)	59/110 (53.6)
[17]	Italy (n = 21)	6/21 (28.6)	6/6 (100)	0/6 (0)	NA	NA
[15]	Italy (n = 41)	21/41 (51.2) ^f^	20/21 (95.2)	0/21 (0)	7/10 (70)	8/20 (40)
[71]	Italy (n = 67)	29/67 (43.3) ^f^	28/29 (96.6)	0/29 (0)	2/3 (66.7)	18/39 (46.2)
[59]	Japan (n = 104)	68/104 (65.4)	66/68 (97.1)	2/68 (2.9)	9/28 (32.1)	40/50 (80)
[60]	Japan (n = 11)	9/11 (81.8)	9/9 (100)	NA	NA	NA
[54]	Japan (n = 11)	4/11 (36.4)	3/4 (75)	1/4 (25)	0/1 (0)	NA
[62]	Japan (n = 125)	85/125 (68)	83/85 (97.6)	2/85 (2.4)	14/27 (51.9)	59/75 (78.7)
[57]	Japan (n = 36)	21/36 (58.3)	21/21 (100)	0/21 (0)	NA	NA
[58]	Japan (n = 36)	23/36 (63.9)	23/23 (100)	NA	NA	NA
[19]	Japan (n = 374)	224/374 (59.9) ^f^	208/224 (92.9)	9/224 (4)	40/107 (37.4)	104/139 (74.8)
[61]	Japan (n = 97)	53/97 (54.6)	52/53 (98.1)	1/53 (1.9)	8/21 (38.1)	21/30 (70)
[54]	Lesotho (n = 2)	1/2 (50)	0/1 (0)	1/1 (100)	1/2 (50)	NA
[54]	Mozambique (n = 6)	4/6 (66.7)	2/4 (50)	2/4 (50)	3/5 (60)	NA
[54]	South Africa (n = 2)	1/2 (50)	1/1 (100)	0/1 (0)	1/2 (50)	NA
[63]	South Korea (n = 105)	41/105 (39)	39/41 (95.1)	2/41 (4.9)	23/78 (29.5)	5/6 (83.3)
[76]	South Korea (n = 160)	46/160 (28.8)	32/46 (69.6)	14/46 (30.4)	19/58 (32.8)	3/5 (60)
[64]	South Korea (n = 205)	57/205 (27.8)	54/57 (94.7)	3/57 (5.3)	32/138 (23.2)	7/16 (43.8)
[15]	Spain (n = 9)	6/9 (66.7)	6/6 (100)	0/6 (0)	1/1 (100)	4/5 (80)
[54]	Swaziland (n = 1)	0/1 (0)	0/1 (0)	0/1 (0)	0/1 (0)	NA
[65]	Taiwan (n = 195)	57/195 (29.2)	54/57 (94.7)	3/57 (5.3)	27/121 (22.3)	24/50 (48)
[54]	Transkei (n = 4)	2/4 (50)	2/2 (100)	0/2	0/1 (0)	NA
[69]	USA (n = 61)	27/61 (44.3)	26/27 (96.3)	1/27 (3.7)	4/15 (26.7)	10/16 (62.5)
[68]	USA (n = 70)	50/70 (71.4)	49/50 (98)	1/50 (2)	0/7 (0)	36/40 (90)
[19]	USA (n = 89)	33/89 (37.1)	31/33 (93.9)	2/33 (6.2)	2/13 (15.4)	20/51 (39.2)
	Total (n = 4133)	2034/4133 (49.2)	1899/2035 (93.3)	99/2035 (4.9)	369/1167 (31.6)	570/861 (66.2)

^a^: Frequency of *TERTp* mutations in the total analyzed samples. ^b^: Frequency of C228T in *TERTp*-mutated samples. ^c^: Frequency of C250T in *TERTp*-mutated samples. ^d^: Frequency of *TERTp* mutations in HBV-positive samples. ^e^: Frequency of *TERTp* mutations in HCV-positive samples. ^f^: *TERTp* mutations other than C228T and C250T were also included. NA, not available.

## 5. Wnt/β-Catenin Signaling

The Wnt/β-catenin signaling pathway is a highly conserved cell communication system that plays a crucial role in embryonic development, cell proliferation, and differentiation. Aberrations in this pathway have been implicated in various diseases, including cancer. The Wnt pathway was first discovered through its role in embryogenesis, where it regulates the fate of cells, cell migration, and organogenesis [77]. In adults, it maintains homeostasis in tissues such as the intestine, skin, bone, and liver [78].

The Wnt pathway can be broadly divided into canonical and non-canonical branches. The canonical pathway, also known as the Wnt/β-catenin pathway, is primarily responsible for regulating gene transcription. In the absence of Wnt ligands (Wnt signaling OFF), β-catenin is continuously degraded by a destruction complex composed of axis inhibitor protein (AXIN), adenomatous polyposis coli (APC), glycogen synthase kinase 3 (GSK-3), and casein kinase 1 (CK1). This complex facilitates the phosphorylation of β-catenin, marking it for ubiquitination and subsequent proteasomal degradation. When Wnt ligands bind to the Frizzled family receptors and co-receptors such as LRP5/6 (Wnt signaling ON), the destruction complex is inhibited. This leads to the stabilization and accumulation of β-catenin in the cytoplasm, which then translocates into the nucleus. Once in the nucleus, β-catenin acts as a co-activator of transcription factors [79,80].

β-catenin is a multifunctional protein encoded by the *CTNNB1* gene, located on chromosome 3p22.1. Structurally, β-catenin comprises an N-terminal domain, a central armadillo repeat domain, and a C-terminal transactivation domain. The central domain contains 12 armadillo repeats, which are crucial for protein–protein interactions. Functionally, β-catenin plays a dual role in cell–cell adhesion and gene transcription regulation. In cell adhesion, β-catenin links cadherins to the actin cytoskeleton, maintaining cell integrity and tissue architecture [81]. Beyond its structural role, β-catenin is pivotal in the regulation of gene expression. When translocated to the nucleus, it associates with T-cell factor/lymphoid enhancer factor (TCF/LEF) transcription factors to modulate the transcription of target genes involved in critical cellular processes such as proliferation, differentiation, and survival [82,83,84]. Additionally, β-catenin interacts with *TERT*, enhancing its transcription and thereby playing a role in cellular immortalization and oncogenesis [36]. The dysregulation of β-catenin, often due to mutations in the *CTNNB1* gene, can lead to its constitutive activation, contributing to the pathogenesis of various diseases, including cancer [85]. Additionally, β-catenin crosstalks with other key signaling pathways, such as Notch, Hedgehog, and Hippo, forming a complex regulatory network that promotes the malignant phenotype of cancer cells [86]. This extensive involvement in various cellular processes underscores the significance of the Wnt/β-catenin pathway in cancer biology.

## 6. Wnt/β-Catenin in HCC

In HCC, the Wnt/β-catenin pathway is frequently activated, playing a pivotal role in tumor development and progression. Genetic alterations affecting this pathway are commonly observed in HCC, underscoring its significance as a critical driver of hepatocarcinogenesis [15]. The activation of the Wnt/β-catenin pathway in HCC is often attributed to mutations in *CTNNB1*. These mutations prevent the degradation of β-catenin, leading to its accumulation and the activation of Wnt target genes. The resultant accumulation of β-catenin not only promotes the proliferation of cancer cells but also enhances their resistance to apoptosis and their capacity to invade surrounding tissues and form metastases [87]. Mutations in β-catenin in HCC are particularly common in exon 3, which encodes a region crucial for the regulation of β-catenin stability. The most frequent mutations occur at key residues that are normally phosphorylated by GSK-3. These amino acid changes disrupt the phosphorylation sites, preventing β-catenin degradation and leading to its stabilization and accumulation [88].

Regarding etiology, *CTNNB1* mutations are notably more frequent in HCV-associated and non-viral HCCs (e.g., alcohol-related and MASLD) than in HBV-related HCCs, although reported frequencies vary across studies depending on the characteristics of the analyzed cohorts [13,59]. While *CTNNB1* mutations represent a critical driver of hepatocarcinogenesis, their prognostic implications in established HCC remain controversial. Some studies suggest that *CTNNB1* mutations correlate with less aggressive tumor behavior, reduced invasiveness, lower serum alpha-fetoprotein (AFP) levels, and better-differentiated HCC [89,90,91]. Conversely, other research associates these mutations with a worse prognosis, including increased small vessel invasion and tumor capsule invasion, or reports no significant impact on overall survival [92,93].

New therapies targeting the Wnt/β-catenin pathway in cancer are being developed, focusing on inhibiting the aberrant signaling that drives tumor growth. Given its role in tumor proliferation, metastasis, and therapy resistance, the Wnt/β-catenin pathway has emerged as an attractive therapeutic target in HCC [85,94]. Several small-molecule inhibitors targeting Wnt/β-catenin signaling have been developed, including LGK974, a Porcupine inhibitor that blocks Wnt ligand secretion, thereby disrupting the activation of the Wnt signaling cascade [95]. Another strategy involves tankyrase inhibitors, which stabilize AXIN, an essential component of the β-catenin destruction complex, leading to enhanced β-catenin degradation [96]. Additionally, inhibitors targeting the interaction between β-catenin and its transcriptional coactivator CBP, such as PRI-724, are being explored for their potential to modulate β-catenin-mediated transcription and suppress oncogenic signaling in cancer cells [97]. In addition to direct inhibition, targeting the Wnt/β-catenin pathway may enhance the efficacy of immunotherapy in HCC. *CTNNB1*-mutated tumors have been shown to exhibit low immune infiltration and resistance to ICIs, particularly anti-PD-1/PD-L1 therapies. This immune exclusion is associated with reduced chemokine expression, leading to a suppressed infiltration of immune cells in HCC [98]. However, preclinical studies suggest that Wnt/β-catenin inhibition may restore immune responsiveness, potentially overcoming resistance to immunotherapy [99]. Efforts to integrate *CTNNB1* mutational status into clinical decision-making are also advancing. In Japan, the NCC OncoPanel initiative, along with other comprehensive genomic profiling (CGP) approaches, have been utilized to analyze genetic alterations in various types of solid tumors, including HCC, and recommend targeted therapies [100]. Recent studies indicate that various mutations, including *CTNNB1*, are being evaluated for their potential role in second-line treatment selection following immunotherapy [101].

## 7. Frequency and Geographic Distribution of *CTNNB1* Exon 3 Mutations in HCC

Published data on the analysis of *CTNNB1* exon 3 mutations in HCC were retrieved from PubMed using the keywords “CTNNB1”, “exon 3”, “mutations”, and “hepatocellular carcinoma”. Table 2 summarizes these studies, highlighting the prevalence of *CTNNB1* exon 3 mutations across different geographic regions and viral etiologies. A total of 40 studies were reviewed. Among the mutation sites identified, the most frequently reported were S45, observed in 58% of the studies, followed by D32 (53%), S37 (50%), T41 (50%), S33 (48%), G34 (40%), and H36 (25%). These mutations affect critical phosphorylation sites involved in β-catenin regulation, potentially leading to its stabilization and accumulation, which is associated with hepatocarcinogenesis. The prevalence of these mutations varied among studies, reflecting differences in study cohorts, geographic distribution, and underlying etiologies. However, the consistent detection of these specific mutations across multiple studies highlights their potential role in HCC pathogenesis (Table 2).

Analysis of 5276 HCC samples revealed that 23.1% harbored *CTNNB1* exon 3 mutations. The reported mutation rates vary significantly, ranging from 0% in Egypt [102] and 2.8% in South Korea [103] to as high as 50% in France [104] and 71.4% in a small cohort from the USA [105]. This variability likely reflects differences in patient populations, environmental exposures, and methodological approaches (Table 2).

**Figure 3:**
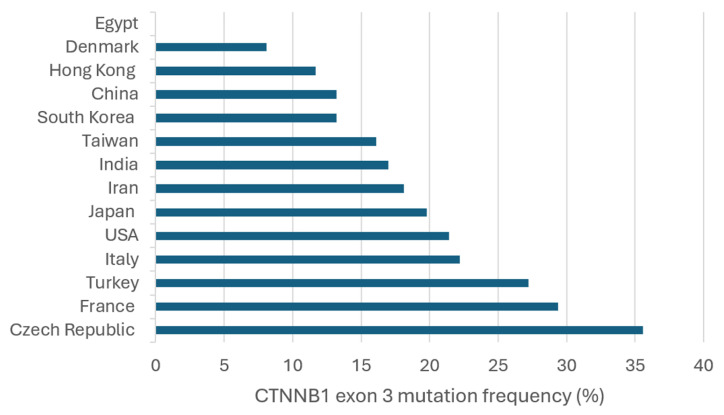
Frequency of *CTNNB1* exon 3 mutations in HCC cases across different geographic regions.

In Europe, mutation frequencies are generally high but vary considerably across studies. France stands out with some of the highest rates, such as 50% [104] and 44.4% [106], though a more recent large-scale study reported a lower prevalence of 27.7% [51]. Schulze et al. (2015) [15] analyzed cases from multiple European countries, including France, Italy, and Spain, and found an overall prevalence of 34.9%, supporting the notion that *CTNNB1* mutations are common in the region. In Italy, Pezzuto et al. (2016) [70] and Tornesello et al. (2013) [107] reported mutation frequencies of 26% and 14.9%, respectively. In the Czech Republic, Ambrozkiewicz et al. (2022) [56] identified a mutation prevalence of 35.6%, while in Turkey, Biterge Süt et al. (2020) [108] found 27.2%. In contrast, Denmark showed a lower frequency of 8.1% [109], suggesting that *CTNNB1* mutations may be less common in Scandinavian HCC cases. In Asia, mutation frequencies tend to be lower overall but show considerable heterogeneity. In Japan, Nishida et al. (2018) [62] reported a prevalence of 24.8%, while Kawai-Kitahata et al. (2016) [59] found 29.8%. However, Hsu et al. (2000) [110] identified a lower prevalence of 13.1%, highlighting variability possibly related to patient selection criteria. Notably, Sekine et al. (2011) [111] and Ki et al. (2016) [60] reported higher frequencies of 42.9% and 45.5%, respectively, indicating that *CTNNB1* mutations can be highly prevalent in certain Japanese cohorts. In China, mutation frequencies range from 5.1% [112] to 24.3% [66]. Taiwan shows similar trends, with Lu et al. (2014) [113] reporting 18.3% and Mao et al. (2001) [90] finding 14.1%. In Hong Kong, Wong et al. (2001) [114] observed a mutation prevalence of 11.7%, while studies from South Korea reported 2.8% [103], 14.6% [63], and 16% [115]. In India, mutation frequencies were 18.8% [116] and 13.3% [117], comparable to values observed in Taiwan and China. In Iran, Javanmard et al. (2020) [118] found a mutation prevalence of 18.1%, aligning with the trend of moderate mutation frequencies in the region. Outside Asia and Europe, data from the USA show moderate-to-high variability. Chianchiano et al. (2018) [68] reported a mutation rate of 15.7%, while Cieply et al. (2009) [119] found 28.1%. However, a small cohort analyzed by Li et al. (2011) [105] showed an exceptionally high mutation rate of 71.4%, which may reflect a highly selected patient population or methodological differences. In Egypt, Hosny et al. (2008) [102] did not detect any *CTNNB1* exon 3 mutations, marking the lowest reported frequency in the dataset (Table 2) (Figure 3).

**Table 2:** Distribution of *CTNNB1* exon 3 mutations in HCC by viral etiology and geographic region.

Refs.	Country/Region	*CTNNB1* Exon 3 (n, %) ^a^	Mutation Sites	HBV+ Mut (n, %) ^b^	HCV+ Mut (n, %) ^c^
[120]	China (n = 156)	15/156 (9.6)	D32G/Y G34E/V S37C T41A S45P	12/109 (11)	NA
[112]	China (n = 39)	2/39 (5.1)	D32N S37F	2/29 (6.9)	NA
[66]	China (n = 70)	17/70 (24.3)	NA	NA	NA
[56]	Czech Republic (n = 59)	21/59 (35.6)	S29S D32V S33C/F/Y G34V S37C/Y T41A S45F/Y G48G E53E V57M	NA	NA
[109]	Denmark (n = 37)	3/37 (8.1)	T41A	NA	1/4 (25)
[102]	Egypt (n = 20)	0/20 (0)	None	NA	NA
[121]	France (n = 137)	26/137 (19)	S23R D32A/G S33C/F/L/S G34R/V I35S H36P S37A/Y T41A/I S45A/F/P	1/42 (2.4)	12/40 (30)
[13]	France (n = 304)	101/304 (33.2)	H24P D32A/G/H/N/V/Y S33A/C/F/P/T/Y G34R/V I35S H36P S37C/F/P T41A/I S45A/F/P/Y	NA	NA
[104]	France (n = 42)	21/42 (50)	D32G/N/Y S33A/C/F/P G34E/R S37A T41A/I S45F/P S45P	3/7 (42.9)	6/9 (66.7)
[106]	France (n = 45)	20/45 (44.4)	D32G S33C/P/Y G34V S37Y T41A S45A/F/P/Y	0/6 (0)	5/8 (62.5)
[51]	France (n = 746)	207/746 (27.7)	NA	NA	NA
[15]	France, Italy, and Spain (n = 235)	82/235 (34.9)	NA	NA	NA
[114]	Hong Kong (n = 60)	7/60 (11.7)	G34V I35S H36P T41A S45F/T	5/48 (10.4)	0/2 (0)
[117]	India (n = 15)	2/15 (13.3)	G32C/S	2/15 (13.3)	NA
[116]	India (n = 32)	6/32 (18.8)	S33T T40P P52H E54A E55Q V57M	6/32 (18.8)	NA
[118]	Iran (n = 105)	19/105 (18.1)	D32G/V S33C H36Q S37C G38R/S/V A39V T41P T42A P44R S45P	9/71 (12.7)	NA
[70]	Italy (n = 127)	33/127 (26)	S33 S37 S45	2/12 (16.7)	29/110 (26.4)
[107]	Italy (n = 67)	10/67 (14.9)	D32H S33A/C G34E/V I35S S37F/Y S45P	0/10 (0)	10/57 (17.5)
[17]	Italy and France (n = 7)	1/7 (14.3)	NA	NA	NA
[59]	Japan (n = 104)	31/104 (29.8)	NA	3/31 (9.7)	19/31 (61.3)
[60]	Japan (n = 11)	5/11 (45.5)	NA	NA	NA
[62]	Japan (n = 125)	31/125 (24.8)	D32N/Y S33C/F/P G34R/V H36P/R S37C/F T41A P44A S45P/F	7/27 (25.9)	21/75 (28)
[111]	Japan (n = 42)	18/42 (42.9)	NA	NA	NA
[110]	Japan (n = 434)	57/434 (13.1)	D32G/N/V/Y S33A/C/P G34E/R/V H36P S37C/F/Y T41A/I S45A/F/P	30/323 (9.3)	23/92 (25)
[122]	Japan and Switzerland (n = 22)	9/22 (40.9)	D32A/G/N/Y S33Y S37P/Y T41A S45F/P	0/22 (0)	9/22 (40.9)
[63]	South Korea (n = 103)	15/103 (14.6)	S29F D32A/G/N/V S33C S37A T41A S45A/F/P	10/76 (13.2)	1/7 (14.3)
[103]	South Korea (n = 36)	1/36 (2.8)	T41A	0/21 (0)	0/4 (0)
[115]	South Korea (n = 81)	13/81 (16)	D32G S33F/P G34R/V H36P S37Y T41A S45F	13/78 (16.7)	0/6 (0)
[113]	Taiwan (n = 115)	21/115 (18.3)	S23N L31P D32G/N/V S33C/P G34E/R/V S37A T41A/I T42I S45P P52L G69E	13/78 (16.7)	5/24 (20.8)
[123]	Taiwan (n = 150)	22/150 (14.7)	NA	NA	NA
[65]	Taiwan (n = 188)	31/188 (16.5)	NA	15/121 (12.4)	14/50 (28)
[124]	Taiwan (n = 214)	32/214 (15)	NA	NA	NA
[90]	Taiwan (n = 262)	37/262 (14.1)	D32 S33 G34 H36 S37 T41 S45	NA	NA
[125]	Taiwan (n = 73)	18/73 (24.7)	NA	NA	NA
[108]	Turkey (n = 360)	98/360 (27.2)	S45P/F/Y D32G/V/N	NA	NA
[119]	USA (n = 32)	9/32 (28.1)	L30Q D32G/V S33C S37F T41F T42P S45P	NA	3/6 (50)
[105]	USA (n = 7) The Netherlands (n = 1) China (n = 1)	5/7 (71.4) 0/1 (0) 1/1 (100)	D32G/H G34V H36P	0 0 1/1 (100)	4/5 (80) 0/1 (0) 0/1 (0)
[68]	USA (n = 70)	11/70 (15.7)	I35S S37C S45F/P/Y	0/7 (0)	9/40 (22.5)
[126]	USA (n = 73)	14/73 (19.2)	D32G S33Y G34E/V T41A S45C/P/Y	NA	NA
[19]	USA and Japan (n = 469)	146/469 (31.1)	Q28R D32G/H/N/V/Y S33A/C/F/P/Y G34E/R/V I35S H36P S37A/C/F/P/Y S45A/C/F/P/T/Y T41A/I T42K	29/108 (26.9)	69/188 (36.7)
	Total (n = 5276)	1218/5276 (23.1)		163/1274 (12.8)	240/782 (30.7)

^a^: Frequency of *CTNNB1* exon 3 mutations in the total analyzed samples. ^b^: Frequency of *CTNNB1* exon 3 mutations in HBV-positive samples. ^c^: Frequency of *CTNNB1* exon 3 in HCV-positive samples. NA, not available.

When comparing viral etiologies, *CTNNB1* exon 3 mutations were identified in 12.8% of HBV-associated HCCs, while the prevalence was considerably higher (30.7%) in HCV-associated cases (Table 2). In Japan, Hsu et al. (2000) [110] reported a mutation frequency of 9.3% in HBV+ cases compared to 25% in HCV+ cases, while Kawai-Kitahata et al. (2016) [59] found an even more striking difference: 9.7% in HBV+ versus 61.3% in HCV+ cases, one of the highest rates observed. Similarly, in Taiwan, mutation frequencies of 12.4% in HBV+ cases and 28% in HCV+ cases were reported [65]. Another study documented frequencies of 16.7% in HBV+ cases versus 20.8% in HCV+ cases [113]. In Europe, the pattern persists. In France, mutation frequencies of 42.9% in HBV+ cases and 66.7% in HCV+ cases were reported [104], while in Italy, a frequency of 16.7% in HBV+ cases versus 26.4% in HCV+ cases was found [70]. In the USA, no mutations were observed in HBV+ cases (0%), compared to 22.5% in HCV+ cases [68], and a significantly higher rate of 80% was reported in HCV+ cases [105] (Table 2). These findings suggest that HCV infection may create a molecular environment more conducive to *CTNNB1* mutations, potentially through chronic inflammation or oxidative stress, while HBV-associated HCC may rely more on alternative oncogenic mechanisms, such as viral DNA integration.

## 8. Interactions Between TERT and β-Catenin in HCC

Mutations in *TERTp* and *CTNNB1* exon 3 are frequently observed in HCC and have been reported to co-occur in some cases, potentially due to complementary oncogenic mechanisms, although this association appears to vary across tumor subtypes and populations. *TERTp* mutations result in telomerase reactivation, allowing hepatocytes to bypass replicative senescence and gain proliferative potential. *CTNNB1* exon 3 mutations, on the other hand, lead to constitutive activation of the Wnt/β-catenin signaling pathway, promoting cellular proliferation and metabolic reprogramming. The convergence of these two alterations may provide a selective advantage in certain HCC subtypes, as *TERT* mutations sustain chromosomal stability and cell immortality, while β-catenin activation enhances tumor initiation and progression [16,127,128]. Some studies suggest that β-catenin transcriptional activity may directly or indirectly influence *TERT* expression, further reinforcing the interplay between these pathways in tumor development [36,37,129]. Additionally, the presence of *CTNNB1* mutations has been associated with a unique molecular subclass of HCC, often characterized by low genomic instability, well-differentiated histology, and persistent telomerase activity, supporting the hypothesis that these mutations may act in concert to sustain tumor progression [128]. While their co-occurrence has been consistently reported in various cohorts, the strength of this association varies across populations and etiological backgrounds, suggesting that additional molecular and environmental factors may modulate this interaction.

To systematically assess the association between *TERTp* and *CTNNB1* exon 3 mutations, several studies have investigated their concordance in HCC. Table 3 provides an overview of the frequency of these mutations across different populations and their statistical correlation (Table 3). Among the studies that reported a significant association, Schulze et al. (2015) [15] analyzed a European HCC cohort, finding 60.5% of tumors with *TERTp* mutations and 34.9% with *CTNNB1* mutations, with a statistically significant correlation (*p* = 0.03). Their analysis also highlighted that *TERTp* mutations were frequent in early tumor stages, while *CTNNB1* mutations were more associated with later stages of hepatocarcinogenesis, reinforcing their role in tumor progression. In a larger trans-ancestry study, Totoki et al. (2014) [19] examined cases from the USA and Japan, reporting 55.5% of tumors with *TERTp* mutations and 31.1% with *CTNNB1* mutations, confirming a highly significant association (*p* < 0.0001). Their findings reinforce the hypothesis that these mutations may co-occur due to shared oncogenic pathways. Similarly, Nault et al. (2013) [13] analyzed French HCC cases and reported a high prevalence of *TERTp* mutations (58.7%) and *CTNNB1* mutations (33.2%), with a significant association (*p* < 0.0001). Their findings indicated that *TERTp* mutations are the most frequent genetic alterations in HCC and occur early in hepatocarcinogenesis, particularly in cirrhotic preneoplastic lesions. *CTNNB1* mutations, on the other hand, are significantly associated with *TERTp* mutations and are involved in tumor progression and malignant transformation, particularly in hepatocellular adenomas undergoing transition to carcinoma. Expanding on these observations, Nault et al. (2020) [51] revisited French HCC cases and confirmed the strong association between these mutations (58.1% *TERTp*, 27.7% *CTNNB1*; *p* < 0.0001). Their study reinforced that *TERTp* mutations are key early events in liver tumorigenesis, while *CTNNB1* mutations are significantly associated with specific molecular subgroups and distinct tumor phenotypes, including well-differentiated tumors and lower AFP levels. Conversely, several studies found no significant correlation between these mutations. Chen et al. (2014) [65] assessed Taiwanese HCC cases, observing 29.2% of tumors with *TERTp* mutations and 16.5% with *CTNNB1* mutations, but no significant correlation between them (*p* = 0.2055). Their analysis revealed that *TERTp* mutations were significantly associated with HCV infection (*p* = 0.0048) and the absence of HBV infection (*p* = 0.0007), suggesting distinct etiological pathways in hepatocarcinogenesis. In contrast, *CTNNB1* mutations did not show a significant correlation with viral infections or other clinical parameters. Lee et al. (2016) [63] investigated South Korean HCC patients, identifying 39% of tumors with *TERTp* mutations and 14.6% with *CTNNB1* mutations, but found no significant correlation between them (*p* = 0.568). Their analysis revealed that *TERTp* mutations were significantly more frequent in HCC cases related to HCV infection (83.3%; *p* = 0.001), whereas *CTNNB1* mutations showed no significant association with viral infections or other clinicopathological factors. Additionally, intratumoral heterogeneity in *TERTp* mutations was observed in recurrent HCC cases, suggesting a potential role in tumor evolution and progression. In a study of Japanese HCC cases, Ki et al. (2016) [60] reported 81.8% *TERTp* and 45.5% *CTNNB1* mutation rates, yet the association was not significant (*p* = 0.4545). Although no statistical significance was reported, the authors observed that all five NAFLD-HCC cases with *CTNNB1* mutations also harbored *TERTp* mutations, suggesting a potential cooperative role in liver carcinogenesis. Yuan et al. (2017) [66] analyzed Chinese HCC cases, reporting 30% *TERTp* and 24.3% *CTNNB1* mutation rates. They observed no significant association between *TERTp* and *CTNNB1* mutations (*p* = 0.535) and suggested that differences in genetic susceptibility or environmental exposures might explain the discrepancy with previous studies that reported a correlation between these alterations. Finally, Pezzuto et al. (2016) [70] examined Italian HCC cases, finding 50.4% *TERTp* and 26% *CTNNB1* mutation rates, but no significant correlation (*p* = 0.4192). The authors suggested that regional differences and variations in etiology, particularly the predominance of HCV-related cases in their cohort, might explain discrepancies with other studies (Table 3).

## 9. Conclusions

The comprehensive review conducted in this study reinforces the critical role of *TERTp* and *CTNNB1* exon 3 mutations in HCC pathogenesis, particularly in the context of viral-related cases. *TERTp* mutations (C228T and C250T) were identified in approximately 49.2% of HCC cases, with higher frequencies observed in European and Japanese populations, regions historically marked by a predominance of HCV over HBV infections. These mutations were more frequently detected in HCV-related HCC. Similarly, *CTNNB1* exon 3 mutations were found in 23.1% of cases, showing a higher prevalence in HCV-associated tumors. The observed co-occurrence of these mutations suggests complementary roles in hepatocarcinogenesis, with telomere maintenance potentially synergizing with aberrant Wnt/β-catenin signaling to promote tumor initiation and progression. This interplay may not only facilitate early tumorigenesis but also contribute to tumor heterogeneity and influence disease progression, which could have implications for treatment resistance and patient outcomes.

Despite the focus of this review on *TERTp* and *CTNNB1* mutations due to their high prevalence and distinct roles in HCC pathogenesis, other molecular alterations, such as *TP53*, *AXIN1*, and *ARID1A* mutations, also play crucial roles in tumor development, progression, and the diversification of tumor heterogeneity. Additionally, some geographic regions remain underrepresented due to the limited availability of published studies. Further research addressing these aspects will provide a more comprehensive global perspective on the molecular epidemiology of HCC and deepen our understanding of its underlying mechanisms.

While metabolic-associated and alcohol-related HCC are increasingly recognized, viral hepatitis remains a major driver of HCC worldwide, with HBV persisting as an incurable infection in millions of individuals and HCV continuing to pose long-term oncogenic risks even after viral eradication. In this context, molecular profiling of *TERTp* and *CTNNB1* mutations may enhance risk stratification and guide personalized surveillance strategies, particularly in patients with viral-associated liver disease. These findings emphasize the need for precision medicine approaches tailored to the molecular and etiological landscape of HCC across different populations. Future studies expanding these analyses to non-viral etiologies will further contribute to a comprehensive understanding of the molecular profile of HCC.

## Figures and Tables

**Table 3:** Frequency and association of *TERT* promoter and *CTNNB1* exon 3 mutations in HCC across studies.

Reference	Country	*TERTp* (n, %)	*CTNNB1* (n, %)	Mutation Correlation
[15]	France, Italy, and Spain	147/243 (60.5)	82/235 (34.9)	Yes (*p* = 0.03)
[51]	France	441/759 (58.1)	207/746 (27.7)	Yes (*p* = 0.0000001)
[13]	France	179/305 (58.7)	101/304 (33.2)	Yes (*p* < 0.0001)
[19]	USA and Japan	257/463 (55.5)	146/469 (31.1)	Yes (*p* < 0.0001)
[66]	China	57/190 (30)	17/70 (24.3)	No (*p* = 0.535)
[63]	South Korea	41/105 (39)	15/103 (14.6)	No (*p* = 0.568)
[60]	Japan	9/11 (81.8)	5/11 (45.5)	No (*p* = 0.4545)
[70]	Italy	64/127 (50.4)	33/127 (26)	No (*p* = 0.4192)
[65]	Taiwan	57/195 (29.2)	31/188 (16.5)	No (*p* = 0.2055)
